# Sclerostin and Its Associations With Bone Metabolism Markers and Sex Hormones in Healthy Community-Dwelling Elderly Individuals and Adolescents

**DOI:** 10.3389/fcell.2020.00057

**Published:** 2020-02-07

**Authors:** Yang Xu, Chao Gao, Jinwei He, Wenqin Gu, Chuntao Yi, Bihua Chen, Qingqing Wang, Feng Tang, Juliang Xu, Hua Yue, Zhenlin Zhang

**Affiliations:** ^1^Shanghai Clinical Research Center of Bone Disease, Department of Osteoporosis and Bone Diseases, Shanghai Jiao Tong University Affiliated Sixth People’s Hospital, Shanghai, China; ^2^Fenglin Community Health Service Center, Shanghai, China; ^3^Longhua Community Health Service Center, Shanghai, China; ^4^Qixian Community Health Service Center, Shanghai, China

**Keywords:** sclerostin, bone metabolism markers, sex hormones, elderly individuals, adolescents, cross-sectional study

## Abstract

Sclerostin is an important regulator of bone mass involving Wnt/β-catenin signaling pathway. We aimed to obtain the profile of serum sclerostin level and explore its associations with bone metabolism markers and sex hormones in healthy community-dwelling Chinese elderly individuals and adolescents. A cross-sectional study was performed in three communities in Shanghai. In all, 861 participants, including 574 healthy elderly individuals, and 287 healthy adolescents, were recruited. The levels of serum sclerostin, procollagen type 1 N-terminal propeptide (P1NP), β-CrossLaps of type I collagen containing cross-linked C-telopeptide (β-CTX), parathyroid hormone (PTH), 25-hydroxyvitamin D [25(OH)D], estradiol (E_2_), testosterone (T), and sex hormone-binding globulin (SHBG) were measured in blood samples from all participants. Median sclerostin level was higher in males than in females and in elderly individuals than in adolescents (elderly males: 54.89 pmol/L, elderly females: 39.95 pmol/L, adolescent males: 36.58 pmol/L, adolescent females: 27.06 pmol/L; both *P* < 0.05). In elderly individuals, serum sclerostin was positively correlated with age (β = 0.176, *P* < 0.001) and T (β = 0.248, *P* = 0.001), but negatively associated to P1NP (β = −0.140, *P* = 0.001). In adolescents, circulating sclerostin was significantly and positively associated with P1NP (β = 0.192, *P* = 0.003). The directions of the association between sclerostin and P1NP were opposite in Chinese elderly individuals and adolescents, which may reflect that sclerostin plays distinct roles in different functional states of the skeleton. Our findings revealed the rough profile of circulating sclerostin level in general healthy Chinese population and its associations with bone metabolism markers and sex hormones, which may provide a clue to further elucidate the cross action of sclerostin in bone metabolism and sexual development.

## Introduction

Sclerostin, an osteocyte-derived Wnt antagonist, inhibits the Wnt/β-catenin signaling pathway and is regarded as the important regulator of bone mass (Van Bezooijen et al., 2004). Activation of the canonical Wnt/β-catenin signaling pathway leads to increased bone formation and bone mass through promoting the maturation of osteoblasts and inhibiting the differentiation of osteoclasts (Glass et al., 2005; Krishnan et al., 2006; Baron and Rawadi, 2007). The clinical importance of sclerostin was initially highlighted by sclerosteosis and van Buchem disease, two sclerosing bone disorders caused by loss-of-function mutations of the *SOST* gene (encoding sclerostin), and further proved by the phenotypical characterization of increased bone mass and bone formation in disease-related animal models (Li et al., 2008; Van Lierop et al., 2011, 2013; Boudin et al., 2017). Based on positive results of experiments in mice and human, two monoclonal antisclerostin antibodies, romosozumab and blosozumab, have been developed as new therapies for osteoporosis and their significant effects in increasing bone mineral density (BMD) and reducing fracture risks were demonstrated (McClung et al., 2014; Recknor et al., 2015; Cosman et al., 2016; Ominsky et al., 2017; Carpenter and Ross, 2019).

Although the biological roles of sclerostin and corresponding benefits of antisclerostin antibodies have been illuminated, much epidemiological data regarding serum sclerostin levels is still unclear. Most researches about sclerostin levels are focused on special groups, and there are few studies on general healthy people from communities (He et al., 2014; Faienza et al., 2017; Hansen et al., 2019; Pekkolay et al., 2019; Singh et al., 2019; Yang et al., 2019). The associations of serum sclerostin to bone metabolism markers in different population show inconsistent results, and only a few studies have been conducted to investigate relations of serum slcerostin to sex hormones (Garnero et al., 2013; Szulc et al., 2013; Yamamoto et al., 2013; Lim et al., 2016). Exploring circulating sclerostin level in healthy population and its association with bone metabolism markers and sex hormones may provide a better understanding into the nature of sclerostin, and elucidate the cross action of sclerostin in bone metabolism and sexual development.

In this cross-sectional study, we recruited community-dwelling elderly individuals and adolescents to acquire the profile of circulating sclerostin level in general healthy Chinese population, investigate the difference of slerostin levels between elderly individuals and adolescents, and explore the associations of serum sclerostin to bone metabolism markers and sex hormones.

## Materials and Methods

### Subjects

**FIGURE 1:**
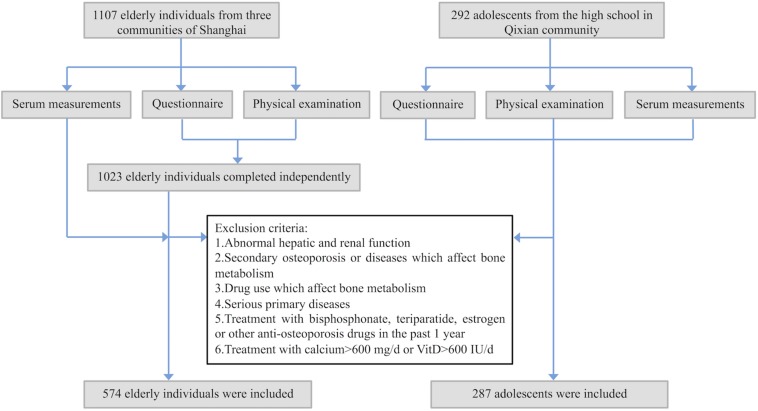
Flowchart for recruiting process of the participants. All participants were evaluated by a questionnaire, physical examination, and routine serum measurements.

This study was approved by the Ethics Committee of the Shanghai Jiao Tong University Affiliated Sixth People’s Hospital. From July to September 2016, we recruited 1107 elderly individuals (aged 65–79 years; men: 365, women: 742) from three communities of Shanghai, i.e., Fenglin, Longhua, and Qixian. All participants were evaluated by a questionnaire, physical examination and routine serum measurements including hepatic and renal function. Only 1023 can complete the questionnaire and physical examination independently. Among the 1023 participants, subjects with the following conditions were excluded: (1) abnormal laboratory measurements, including serum creatinine (Cr), uric acid (UA), alkaline phosphatase (ALP), and alanine aminotransferase (ALT); (2) secondary osteoporosis or diseases that could affect bone metabolism, including osteogenesis imperfecta, Paget’s disease of bone, diabetes mellitus, primary hyperparathyroidism, rheumatoid arthritis or malignant tumors; (3) drug use that could affect bone metabolism, including the use of synthetic steroid hormones, epinephrine or anticonvulsive drugs; (4) serious primary diseases affecting the cardiovascular, pulmonary, hematopoietic, gastrointestinal, renal or nervous systems or mental state; (5) treatment with bisphosphonate, teriparatide, estrogen or other anti-osteoporosis drugs in the past 1 year; and (6) treatment with calcium >600 mg/d or VitD > 600 IU/d. Finally, 574 participants (men: 164, women: 410) were found to be in good health and enrolled in this study (Figure 1).

Moreover, to fill in the gap of researches on sclerostin in Chinese adolescents, and explore the role of sclerostin in different physiological states, such as different age, bone metabolism status and sex hormone levels, 292 adolescents (aged 14–18 years; men: 143, women: 149) were recruited from Fengxian high school in the Qixian community. After the same screening process as in elderly participants, 287 adolescents in good health (men: 140, women: 147) were enrolled in this study (Figure 1). All 861 participants were of Han ethnicity and provided written informed consent.

### Biochemical Measurement

Blood samples were obtained from fasting participants in the morning from 8:00 to 10:00 and stored at −80°C. The following markers of bone metabolism and sex hormone levels were measured by electrochemiluminescence immunoassay: procollagen type 1 N-propeptide (P1NP), β-CrossLaps of type I collagen containing cross-linked C-telopeptide (β-CTX), intact parathyroid hormone (PTH), and 25-hydroxyvitamin D [25(OH)D], estradiol (E_2_), testosterone (T), and sex hormone-binding globulin (SHBG). All markers were measured using the following kits (Roche Diagnostics GmbH): total P1NP kit for P1NP, β-Crosslaps kit for β-CTX, PTH kit for PTH, vitamin D total kit for 25(OH)D, estradiol III kit for E_2_, testosterone II kit for T and SHBG kit for SHBG. The free androgen index (FAI) was calculated as the percentage ratio of testosterone to SHBG values (Vermeulen et al., 1999). Serum sclerostin was measured by enzyme-linked immunosorbent assay (ELISA) using polyclonal goat anti-human sclerostin as the capture antibody, biotin-labeled monoclonal mouse anti-human sclerostin as the detection antibody, and horseradish peroxidase-streptavidin and tetramethylbenzidine for the chromogenic reaction (Biomedica Medizinprodukte GmbH and Co., KG). The intra- and interassay CVs were 4–6% and 5–7%, respectively.

### Statistical Analysis

Normally distributed data are presented as the mean ± standard deviation (SD), while non-normally distributed data are presented as the median and interquartile range. Mann–Whitney *U* test was used to determine differences of the baseline characteristics between males and females, elderly individuals and adolescents. Spearman correlation analysis was performed between sclerostin and other factors; the Spearman correlation coefficients for sex, age, BMI, bone metabolism markers and sex hormones were calculated to determine which factors would be included in the next regression analysis. Then regression analysis was used to explore associations between sclerostin and each factor, and multiple linear stepwise regression analysis was adopted to adjust for sex, age and BMI. All analyses were carried out using SPSS software, version 23 for Mac (IBM, Chicago, United States), and *P* values less than 0.05 were considered significant.

## Results

### General Characteristics of the Study Population

The basic characteristics of the 861 participants were presented in Table 1. Elderly males had a median serum sclerostin level of 54.89 pmol/L (interquartile range: 43.34–69.88 pmol/L), while elderly females had a lower median sclerostin level of 39.95 pmol/L (interquartile range: 30.65–52.00 pmol/L). Similarly, the median sclerostin level of 36.58 pmol/L (interquartile range: 29.02–44.49 pmol/L) in adolescent males was higher than the median level of 27.06 pmol/L (interquartile range: 20.28–34.45 pmol/L) in adolescent females. The serum sclerostin level was significantly higher in males than in females (*P* < 0.05), and in elderly individuals than in adolescents (*P* < 0.05).

**TABLE 1:** Basic characteristics of the 861 participants.

	Elderly individuals		Adolescents		*P* value
	Males	Females	Males	Females	
No. of subjects	164	410	140	147	
Age, y	70(66–73)	70(67–77)	16(16–17)	16(16–17)	<0.05^cd^
BMI, kg/m2	24.22(22.49–26.45)	23.72(21.64–26.17)	20.99(19.23–22.76)	20.64(19.36–23.01)	<0.05^cd^
P1NP, ng/mL	45.28(36.89–57.85)	58.56(45.66–74.15)	181.80(141.45–277.68)	101.60(82.97–125.60)	<0.05^abcd^
β-CTX, ng/mL	0.32(0.23–0.43)	0.42(0.30–0.53)	0.44(0.34–0.62)	0.25(0.20–0.33)	<0.05^abcd^
25(OH)D, ng/mL	26.00(22.00–31.05)	23.81(19.00–30.00)	18.53(14.22–23.38)	19.66(15.26–25.82)	<0.05^acd^
PTH, ng/L	38.15(29.28–48.80)	38.50(30.60–52.30)	39.00(27.68–49.58)	32.00(23.30–40.20)	<0.05^bd^
T, nmol/L	17.34(13.16–22.99)	0.59(0.38–0.91)	15.52(12.75–18.44)	1.09(0.76–1.43)	<0.05^abcd^
SHBG, nmol/L	59.60(42.90–76.38)	73.30(51.05–99.10)	27.40(21.08–34.35)	49.40(33.40–72.40)	<0.05^abcd^
FAI,%	31.25(25.91–36.64)	0.82(0.45–1.55)	58.37(48.27–69.74)	2.21(1.24–3.70)	<0.05^abcd^
E_2_, pmol/L	119.50(89.83–148.95)	46.17(31.02–72.87)	82.44(66.15–97.47)	186.40(114.10–353.60)	<0.05^abcd^
Sclerostin, pmol/L	54.89(43.34–69.88)	39.95(30.65–52.00)	36.58(29.02–44.49)	27.06(20.28–34.45)	<0.05^abcd^

*BMI, body mass index; P1NP, procollagen type 1 N-terminal propeptide; β-CTX, β-CrossLaps of type I collagen containing cross-linked C-telopeptide; 25(OH)D, 25-hydroxyvitamin D; PTH, parathyroid hormone; T, testosterone; SHBG, sex hormone-binding globulin; FAI, free androgen index; E_2_, estradiol. Non-normally distributed data are shown as the median (interquartile range). ^*a*^*P* < 0.05 when comparing the males vs. females in elderly individuals. ^*b*^*P* < 0.05 when comparing the males vs. females in adolescents. ^*c*^*P* < 0.05 when comparing the elderly males vs. the adolescent males. ^*d*^*P* < 0.05 when comparing the elderly females vs. the adolescent females.*

### Correlations of Serum Sclerostin With Bone Metabolism Markers and Sex Hormones

The correlations between serum sclerostin and bone metabolism markers and sex hormones were studied in both elderly individuals and adolescents (Table 2). For elderly individuals, serum sclerostin was negatively correlated with sex, P1NP, β-CTX, and PTH (*r* = −0.343, *r* = −0.212, *r* = −0.116, and *r* = −0.128, respectively; *P* < 0.05), but positively correlated with age, 25(OH)D, T, FAI and E_2_ (*r* = 0.109, *r* = 0.143, *r* = 0.363, *r* = 0.330, and *r* = 0.257, respectively; *P* < 0.05). For serum sclerostin in adolescents, the negative correlations with sex, 25(OH)D, SHBG, and E_2_ (*r* = −0.382, *r* = −0.120, *r* = −0.125, and *r* = −0.283, respectively; *P* < 0.05), and positive correlations with P1NP, β-CTX, PTH, T, and FAI (*r* = 0.333 *r* = 0.328, *r* = 0.156, *r* = 0.349, and *r* = 0.276, respectively; *P* < 0.05) were found.

**TABLE 2:** Correlation analyses of serum sclerostin and other parameters in elderly individuals and adolescents.

	Elderly individuals		Adolescents	
Variables	*r*	*P* value	*r*	*P* value
Sex	–0.343	**<0.001**	–0.382	**<0.001**
Age, y	0.109	**0.011**	0.028	0.650
BMI, kg/m^2^	0.005	0.911	0.003	0.955
P1NP, ng/mL	–0.212	**<0.001**	0.333	**<0.001**
β-CTX, ng/mL	–0.116	**0.007**	0.328	**<0.001**
25(OH)D, ng/mL	0.143	**0.001**	–0.120	**0.048**
PTH, ng/L	–0.128	**0.003**	0.156	**0.010**
T, nmol/L	0.363	**<0.001**	0.349	**<0.001**
SHBG, nmol/L	–0.055	0.210	–0.125	**0.039**
FAI,%	0.330	**<0.001**	0.276	**<0.001**
E_2_, pmol/L	0.257	**<0.001**	–0.283	**<0.001**

*BMI, body mass index; P1NP, procollagen type 1 N-terminal propeptide; β-CTX, β-CrossLaps of type I collagen containing cross-linked C-telopeptide; 25(OH)D, 25-hydroxyvitamin D; PTH, parathyroid hormone; T, testosterone; SHBG, sex hormone-binding globulin; FAI, free androgen index; E_2_, estradiol. Significant values (*P* < 0.05) are presented in bold.*

### The Multiple Regression Analyses Between Sclerostin and the Correlated Factors

Factors associated with the serum sclerostin level were explored using multiple linear regression analysis (Table 3). In elderly individuals, serum sclerostin was positively associated with age (β = 0.176, *P* < 0.001), and after adjusting for sex, age and BMI, it was still positively associated with T (β = 0.248, *P* = 0.001), but negatively associated to P1NP (β = −0.140, *P* = 0.001). In adolescents, serum sclerostin was significantly and positively associated with P1NP (β = 0.192, *P* = 0.003). After adjusting for sex, age and BMI, the association still existed. There were no associations between serum sclerostin and other bone metabolism markers or sex hormones.

**TABLE 3:** Multiple linear regression analyses between sclerostin and the correlated factors.

	Elderly individuals				Adolescents			
Variables	Standardized β-coefficients	*P* Value	Standardized β-coefficients^a^	*P* Value^a^	Standardized β-coefficients	*P* Value	Standardized β-coefficients^a^	*P* Value^a^
Sex	–0.042	0.629			–0.073	0.710		
Age, y	0.176	**<0.001**			0.044	0.444		
BMI, kg/m^2^	–0.026	0.575			0.106	0.103		
P1NP, ng/mL	–0.189	**0.001**	–0.140	**0**.**001**	0.084	0.438	0.192	**0**.**003**
β-CTX, ng/mL	0.082	0.162	0.079	0.161	0.070	0.484	0.055	0.567
25(OH)D, ng/mL	0.086	0.059	0.063	0.142	–0.058	0.323	–0.067	0.247
PTH, ng/L	–0.029	0.515	–0.060	0.158	0.020	0.768	0.018	0.780
T, nmol/L	0.206	**0.036**	0.248	**0**.**001**	0.277	0.051	0.204	0.122
SHBG, nmol/L	–0.070	0.178	–0.069	0.122	–0.014	0.853	–0.006	0.933
FAI,%	0.073	0.395	0.118	0.132	–0.164	0.331	–0.079	0.617
E_2_, pmol/L	0.081	0.182	0.037	0.526	–0.128	0.051	–0.121	0.063

*BMI, body mass index; P1NP, procollagen type 1 N-terminal propeptide; β-CTX, β-CrossLaps of type I collagen containing cross-linked C-telopeptide; 25(OH)D, 25-hydroxyvitamin D; PTH, parathyroid hormone; T, testosterone; SHBG, sex hormone-binding globulin; FAI, free androgen index; E_2_, estradiol. Significant values (*P* < 0.05) are presented in bold. ^*a*^ Adjusted for sex, age and BMI.*

## Discussion

In this study, the basic profile of serum sclerostin among the healthy community-dwelling Chinese population, including elderly individuals and adolescents, was first revealed. The results showed the circulating sclerostin level was significantly higher in elderly individuals than in adolescents, and serum sclerostin was positively correlated with age in elderly individuals, though the relationship was not found in adolescents. These observations correspond with previous studies (Modder et al., 2011b; Amrein et al., 2012; Coulson et al., 2017). The studies concluded that serum sclerostin levels peak early in life (∼age 10 years in girls and 14 years in boys), decline during the later stages of puberty toward a nadir at the end of puberty, and then increase over the remainder of adult life (Kirmani et al., 2012). According to its inhibitory action in osteoblast function, sclerostin is a potential candidate as the biomarker of bone formation. The increasing sclerostin level with age in elderly suggests the enhanced production may be part of the age-related impairment in bone formation. Thus, the difference of circulating sclerostin between adolescents and elderly reflects functional status of the bone, rather than total bone mass (Tsentidis et al., 2016). In addition, we also found sclerostin in males was higher than in females in both elderly individuals and adolescents. The gender difference in sclerostin levels appeared to be established during puberty, and difference in sex hormones or bone metabolism might be the cause (Kirmani et al., 2012).

Interestingly, we found serum sclerostin was positively correlated with P1NP in adolescents, but inversely associated with P1NP in elderly individuals. Although many studies have been performed to explore the correlations between sclerostin and bone metabolism markers, the results are controversial. Some studies have observed that serum P1NP and β-CTX are inversely associated with sclerostin in elderly subjects, while others have revealed no associations between them (Ardawi et al., 2011; Modder et al., 2011b; Costa et al., 2013; Durosier et al., 2013; Yamamoto et al., 2013; Catalano et al., 2014; Neumann et al., 2014). In adolescents, sclerostin levels were positively associated with P1NP and β-CTX (Kirmani et al., 2012). Even in adolescents with T1DM, sclerostin levels were still positively correlated with logCTX and logOsteocalcin (Tsentidis et al., 2016). The relations between scletostin and bone turnover markers were exactly opposite in elderly individuals and adolescents. In adolescents, young skeleton is submitted to growth where bone turnover might be activated to shift in a state of more gain in bone formation rather than resorption, and the metabolism is differentiated from that of elderly people (Tsentidis et al., 2016). Aline et al. proposed that the direction of the association between sclerostin and bone markers depends upon whether bone turnover is increased or decreased (Costa et al., 2013). The finding may reflect that sclerostin might play distinct roles in different functional states of the skeleton, the growing state in adolescents and the reduction of bone formation in elderly individuals (Tsentidis et al., 2016).

Regarding associations between sclerostin and sex hormones, previous studies showed that estrogen, but not testosterone, reduces circulating sclerostin levels, and the higher estrogen levels present in girls following the onset of puberty lead to their lower sclerostin levels that persist during adult life (Modder et al., 2011a; Kirmani et al., 2012). Consistent with this, Mohammed-Salleh et al. found sclerostin was negatively correlated with E_2_ in both pre- and postmenopausal women, and Faryal et al. found significantly negative association between sclerostin and the free estrogen index in postmenopausal women (Mirza et al., 2010; Ardawi et al., 2011). However, in our study, we only found sclerostin was positively associated with T in elderly individuals, while no associations between sclerostin and E_2_ were found. Since T was previously thought to be an anabolic factor for bone, the correlations between sclerostin and T and the biological effects of T on the circulating sclerostin level need to be determined in future studies (Riggs et al., 2002).

Some limitations of this study should be noticed. First, many other important variables were lacked, such as BMD, which is closely related to sclerostin. A positive correlation has been reported between sclerostin and BMD in many studies, however, this study did not correct for the effect of BMD. Second, the cross-sectional nature of this study may limit the statistical inference to non-causal results. Thirdly, the small sample size may have decreased the power of the statistical analyses.

In conclusion, we are the first to reveal the rough profile of the serum sclerostin level in healthy Chinese elderly individuals and adolescents from communities, and explore the associations between sclerostin with bone metabolism markers and sex hormones. The results showed the circulating sclerostin level was significantly higher in elderly individuals than in adolescents, and in males than in females. Additionally, opposite directions of the association between sclerostin and P1NP in elderly individuals and adolescents, and positive correlations between sclerostin and T in elderly individuals were revealed. Our findings may provide a clue to elucidate the cross action of sclerostin in bone metabolism and sexual development.

## Data Availability Statement

The raw data supporting the conclusions of this article will be made available by the authors, without undue reservation, to any qualified researcher.

## Ethics Statement

The studies involving human participants were reviewed and approved by the Ethics Committee of the Shanghai Jiao Tong University Affiliated Sixth People’s Hospital. Written informed consent to participate in this study was provided by the participants’ legal guardian/next of kin.

## Author Contributions

YX and CG conducted the study and analyzed the data. YX wrote the draft of the manuscript. JH, WG, CY, BC, QW, FT, and JX recruited the subjects. ZZ and HY supervised the study and revised the manuscript. All authors read and approved the final manuscript.

## Conflict of Interest

The authors declare that the research was conducted in the absence of any commercial or financial relationships that could be construed as a potential conflict of interest.
